# Effects of step width and gait speed on the variability of mediolateral control in the head and trunk during gait

**DOI:** 10.1371/journal.pone.0320652

**Published:** 2025-04-21

**Authors:** Yu Takada, Tomoaki Atomi, Takayuki Yagi, Shoma Yamamoto, Masao Tomita, Miho Shimizu, Yoriko Atomi

**Affiliations:** 1 Department of Rehabilitation, Uno Hospital, Okazaki, Aichi, Japan; 2 Material Health Science, Graduate School of Engineering, Tokyo University of Agriculture and Technology, Koganei, Tokyo, Japan; 3 Department of Physical Therapy, Faculty of Health Sciences, Kyorin University, Mitaka, Tokyo, Japan; 4 Department of Rehabilitation, Faculty of Health Sciences, Fujita Health University, Toyoake, Aichi, Japan; University of Tehran, IRAN, ISLAMIC REPUBLIC OF

## Abstract

Step width is a parameter that determines the size of the base of support (BOS) during gait. Further, it is related to the control of center of mass (COM) and trunk movements and gait speed. The current study aimed to validate the effect of conditioning using combined step width (narrow and wide) and gait speed (normal [4.5 km/h] and slow [2.2 km/h]) on the segmental control of the head, thorax, and pelvis with respect to the BOS. The behavior of the head, thorax, and pelvis of 17 healthy adult participants were measured during treadmill walking using a three-dimensional motion analysis system. If the step width was narrow, the whole body segment with a high contribution to COM under narrow BOS conditions was more likely to have a high variability. However, the mediolateral direction behavior was small. On the contrary, if the step width was wide, the whole body segment with a high contribution to COM under wide BOS conditions was more likely to have a low variability. Nevertheless, the mediolateral direction behavior was large. Regarding the intersegmental association, particularly if the step width was narrow and the gait speed was normal, the head showed highly controlled movements with minimal displacement and increased fine-tuning. The thorax displayed significant importance in maintaining trunk stability, operating within a larger range of mediolateral displacement compared to the head and pelvis, under three conditions, except if the step width was narrow and the gait speed was normal. The study underscores the significant impact of both step width and gait speed on the control and stability of high-mass body segments during gait. It suggests that narrow step widths necessitate advanced control strategies, while wide step widths promote simpler, compensatory mechanisms, especially relevant in clinical contexts.

## Introduction

Humans can control the center of mass (COM) located high within a narrow base of support (BOS) [1]. Dynamic balance control is exercised during gait, particularly at the frontal plane [2]. The stability of balance control is represented by the association between the COM and BOS, which is known as the margin of stability (MOS) [3,4], where MOS, or stability, is ensured if the distance between moving COM and the outer edge of BOS is maintained [3–5]. In particular, the mediolateral stability at the frontal plane can be evaluated by the positional relationship of the COM relative to step width, which is the mediolateral width of the BOS, and this is important for determining stability [4]. By contrast, COM refers to the center of mass of the whole body, with the head and trunk representing 50%–60% of the body mass [6,7]. Hence, these structures have a significant contribution to COM control. Moreover, the head–trunk are structurally highly segmented and are segmented controlled [8]. Therefore, the concept is quite simple, when considering strategies related to dynamic stability at the frontal plane during gait, the control of head–trunk relative to the mediolateral width of BOS (step width) based on the concept of stability assessed by MOS should be analyzed. Simultaneously, the segmentation from the head to the trunk during gait must be considered [9].

Several reports have assessed control from the head to the trunk during gait. The head decreases vibration during gait [9] to facilitate integrated control, including visual and vestibular information [10]. In addition, the functional characteristics of the head influence posture reflexes based on its motion and orientation [11]. Considering the presence of reflexive posture control mechanisms [12], the stability of the head is important. The trunk is structurally segmented. That is, it is controlled in a multi-segmental and flexible manner, not by a single segment [13–20]. Based on recent studies, the use of segmented evaluation for accurately assessing trunk movement has been emphasized [13]. Hence, differences in control patterns can be identified [14,21], and distinctive movements during gait can be evaluated [16–18]. Further, the unique pelvic movement pattern in humans during gait (pelvic drop on the swing side) is still robust and unchanged even with variations in the mediolateral width of the BOS [22].

These mediolateral movements from the head to the trunk are related to the mediolateral width of the BOS [23–25]. In particular, several reports have shown that restricting the movement of the trunk voluntarily or limiting segmented movements from the thoracic to the lumbar region using trunk orthoses results in a decrease in step width along with the movement distance and velocity of the trunk [24,25]. Conversely, reducing step width decreases the movement distance and velocity of the COM [26]. Therefore, segmented and coordinated control from the head to the trunk is a strategy for dynamic stability in gait relative to the mediolateral width of the BOS [22]. However, only a few studies have examined the association between segmented control of the head and trunk and step width.

Adjusting the movements of multiple segments during gait is an advanced balancing skill [27]. In individuals with functional impairments, adjusting the association between multiple segments such as the head, thorax, and pelvis within a narrow mediolateral BOS during gait imposes high load on the control system. However, to the best of our knowledge, there are no reports examining the variability of the mediolateral association between the head, thorax, and pelvis from the perspective of movement variability in cases where the mediolateral width of the BOS is narrow or wide during gait. This is because operational load is reflected in the variability of the overall movement and its trajectory [28].

In the overview of previous studies, several studies have examined segmental movement control from the head to the trunk during walking, such as the report that trunk sway is an indicator of stability in clinical balance tests [29]; a report that in the walking of the elderly, rotation of the thoracic region occurs as a compensation for mobility loss in the pelvis [30]; and a report that kinematic changes due to a decrease in the range of motion of the trunk are observed earlier than changes in walking speed as an effect of aging on walking [31]. Furthermore, the ability to control segmental movements of the trunk is impaired in patients with stroke and reduced walking stability [32]. Therefore, clarifying the relationship between walking stability and segmental control of the head to the trunk is important. However, segmental coordinated control of the head to the trunk from the perspective of segmental movement variability during walking has not been reported.

In addition, gait speed affects various parameters during gait. Moreover, when conducting gait studies, the influence of gait speed on gait patterns should be considered [33,34]. Healthy adults have a slow gait speed and an increased mediolateral width of the BOS [35]. Further, they experience changes in the variability and stability of the trunk (thoracic) movement [36]. Therefore, a slow gait speed affects mediolateral balance control. However, to the best of our knowledge, there are no reports examining segmented control from the head to the trunk with respect to the mediolateral width of the BOS if the gait speed is equivalent to the average speed of healthy adults and the speed has not decreased.

Therefore, this study aimed to elucidate the effect of variations in stride width or gait speed on segmented control from the head to the trunk with respect to the mediolateral width of the BOS. Based on previous studies, we hypothesized that narrowing the stride width increases the variability in the behavior and control of the head, thorax, and pelvis and that this effect becomes more pronounced with slower gait speeds. Particular attention is paid to the variability in mediolateral movement distance in the head, thorax, and pelvis, focusing on intersegmental comparisons. To achieve this, healthy young adults were included as subjects in treadmill gait conditions with different step widths and gait speeds, the mediolateral movement distance of the head, thoracic spine, and pelvis will be measured using a three-dimensional motion analysis system. The coefficient of variation was used to evaluate the variability in the reproducibility of the trajectory of each segment. Therefore, the significance of this study is that it provides basic data for investigating the relationship between reduced ability to coordinate segmental control of the head to trunk and walking ability caused by aging, stroke, and spinal lesions, among others.

## Methods

### Participants

This study included 17 healthy young people. Among them, 5 were men and 12 women. To examine the influence of step width variability [23,37], we used G * Power 3.1 to calculate sample size, setting an α level of 0.05, a statistical power (1 - β) of 0.8, and a Cohen’s d of 0.8. The inclusion criteria were as follows: healthy individuals with no history of central nervous system or orthopedic diseases and those aged ≤ 39 years (this age range is considered young in Japan). The exclusion criteria were as follows: individuals who complained of physical symptoms (e.g., dizziness, vertigo, and pain) immediately before the measurement task (i.e., walking). The study protocol was approved by the Ethics Committee of Uno Hospital (approval no. UE201801) and Tokyo University of Agriculture and Technology (approval no. 210505-0317). This study started on August 1, 2017, with the recruitment of subjects, and data collection was completed by March 31, 2019. This study was performed in accordance with the Declaration of Helsinki. In addition, the participants were informed about the content and risks of the study before their participation. Further, they provided a written informed consent.

### Experimental protocol

**Fig 1:**
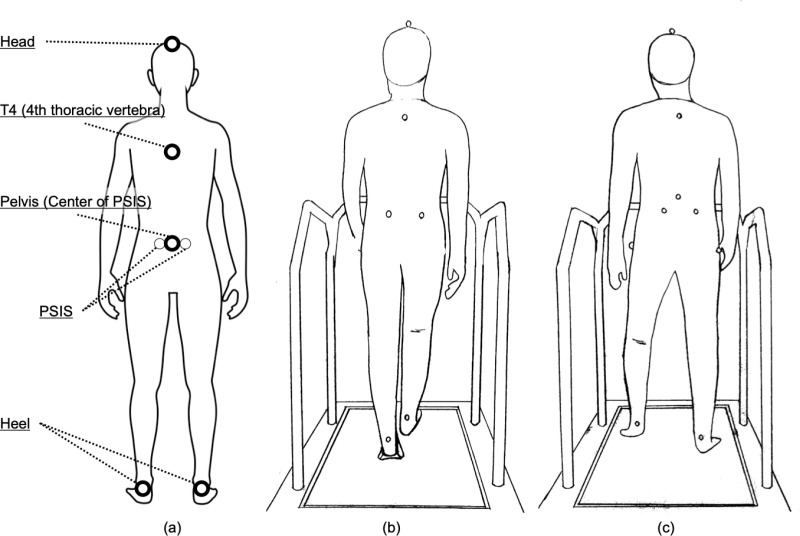
Marker set and walking example. (a) Marker set. Typical example of gait conditions with a (b) narrow and (c) wide step width.

The participants wore measuring clothes, and infrared reflective markers were affixed to each of their respective body parts. The markers were placed in the following areas: the top of the head (head), the fourth thoracic spinous process, the right (Rt) and left (Lt) posterior superior iliac spines, and the Rt and Lt calcaneal eminences (heel). Fig 1a shows the position of the marker. The participants performed a treadmill (Autorunner AR-100, Minato Medical Science Co., Ltd.) walk with bare feet. OptiTrack bar (V120: Trio) (NaturalPoint, Inc.) was the motion capture system used, and the position data of the markers were recorded for approximately 30 s at a sampling rate of 120 fps. The OptiTrack bar is a self-contained system with three built-in cameras and is precalibrated. The camera can detect infrared rays to obtain spatial information about the markers, and the data are acquired using Motive, a special software. This system has a high accuracy and is also used to validate the accuracy of other motion capture systems [38].

Four patterns of conditions were prepared according to the step width and gait speed. Measurements were performed randomly in each trial. As shown in Figs 1(b), 1(c), a narrow base (NB) and a wide base (WB), which are two patterns of the step width, were prepared. The participants received verbal instructions about the change in the step width. In NB, the participants were instructed to narrow the distance between the right and left calcaneus without adopting the scissors gait. In WB, the participants were instructed to walk with the center of the calcaneus always outside the vertical line drawn from the ipsilateral anterior superior iliac spine. In this study, setting the conditions larger than the step width for normal walking was necessary. Because the pelvic width of healthy adults has been reported to be approximately 24.0 ±  1.6 cm [39], we believe that we could set the condition of a wide base by inducing a step width wider than the pelvic width. Two gait speed patterns were prepared: 4.5 km/h (normal) and 2.2 km/h (slow). Between the two patterns, 4.5 km/h was selected as the average gait speed for healthy adults based on the gait speed used in previous studies [40]. Then, a gait speed of 2.2 km/h is associated with an increased risk of falls in elderly people [41]. In contrast, the minimum walking speed of the elderly has been reported to be 2.2–2.5 km/h [42,43], and it has been emphasized that a walking speed of approximately 2.2 km/h (0.6 m/s) may be among the boundaries for expressing mobility among the elderly living in the community [44]. We considered it appropriate to set a standard walking speed of 4.5 km/h and a slow walking speed of 2.2 km/h as speeds that clearly distinguish between high and low mobility cases. To ensure accuracy, one method of defining step width is by placing the participants at the center of the foot on a specified line [45]. However, in this experiment, the participants were only provided with verbal instructions, referring to previous reports [46]. This is because the participants’ head may not behave naturally if the eyes are directed toward the feet to place the feet on the line. Nevertheless, to ensure the accuracy of the experiment, the participants practiced the measurement conditions in advance. The actual measurements were taken after the practice session. The measurement was started after having practiced enough to be able to perform the oral instructions provided.

### Data processing

**Fig 2:**
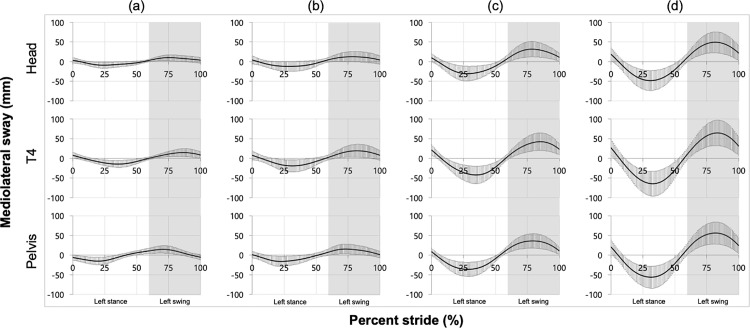
Time series data of the head, T4 level, and pelvis under each measurement condition. Condition: (a) narrow and 4.5km/h, (b) narrow and 2.2km/h, (c) wide and 4.5km/h, (d) wide and 2.2km/h. Black solid lines represent the mean over the entire gait cycle, and error bars indicate ±  SD.

The current analysis only used data from the mediolateral directions. This is because the variability of the trunk during the gait cycle is more likely to appear in the lateral direction than in the motion direction. The analysis sites were the head, T4 level, and pelvic region, which are the main regions of interest. Data processing was performed as follows concerning the report [47] by McGibbon et al. First, the data were low-pass filtered with a fourth-order Butterworth filter with a cutoff frequency of 6 Hz. Next, heel contact was defined as the point at which the heel marker was the lowest. The left heel contact was defined as the start of one walking cycle, and seven consecutive walking cycles were extracted from each data set. Finally, the data from each of the seven walking cycles was divided into one gait cycle, which was used for parameter calculation and statistical analysis. MATLAB R2017b was used for all data processing. Fig 2 plots the mean and standard deviation (SD) of the time series data for all participants and all walking cycles for each of the four patterns.

### General gait parameters

The mean values of stride time (s), step length (mm), and step width (mm) over seven gait cycles were calculated as representative values for each participant in each condition. Stride time was calculated as the time from one left heel contact to the next. Step length was defined as the distance in the sagittal plane from the left (or right) heel contact to the right (or left) heel contact. Step width was defined as the distance in the frontal plane from the left (or right) heel at the left (or right) heel contact to the right (or left) heel at the right (or left) heel contact.

### Definition of parameters related to head and trunk control

#### DIS: Mediolateral displacement.

The distance between the most rightward and leftward points (i.e., the lateral amplitude) was calculated for each walking cycle for the head, T4 level, and pelvic torso segments. The average of the lateral amplitudes over seven walking cycles was considered as the representative value for each participant in each condition, which was defined as the DIS.

#### %DIS: Relative magnitude of DIS to step width (1).

The relative ratio of DIS to step width was calculated by dividing the DIS by the step width for each walking cycle for the head, T4 level, and pelvis. The average value of the relative ratio over seven walking cycles was considered as the representative value for each participant in each condition and was defined as the relative magnitude of DIS to step width. By calculating the magnitude of the DIS of each body segment relative to the step width, the influence of step width can be excluded. Further, the factor of gait speed can be validated. The stability and robustness of each body segment (i.e., the ratio of the DIS of the head and trunk to the width of the BOS) can also be analyzed as follows:


$$\%DISpergaitcycle\left(mm/mm\right)=\;DISpergaitcycle/stepwidthpergaitcycle$$ (1)

#### CV-%DIS: Variability of %DIS (2).

The coefficient of variation (CV) of %DIS was calculated by dividing the SD for seven gait cycles by the mean value. This represents the variability of the stability and robustness of each body segment under each gait condition.


$$CV-\%DIS\left(\%\right)=\left[\left(SDof\%DISoversevengaitcycles\right)/\left(meanof\%DISoversevengaitcycles\right)\right]*100$$ (2)

Couto et al. [48] reported that in a learning process of certain performance, the group with a higher stability exhibited a greater variability in movement. Based on a previous study, a certain degree of movement variability is conducive to performance, which is associated with motor adaptation and learning, thereby indicating a more flexible control [49]. In addition, the CV is not only a variability but also a repeatability [50]. In this study, CV was used as an index of repeatability.

### Statistical analysis

The general parameters in gait were analyzed using two-way analysis of variances (ANOVAs) with SW; step width (NB, WB) and SP; gait speed (Normal, Slow) as factors after confirming normality using the Shapiro–Wilk tests. The parameters related to head and trunk control were analyzed using a three-way within-participant (repeated measures) analysis of variance with SW; step width (NB, WB), SP; gait speed (Normal, Slow), and BS; body segments (head, T4 level, and pelvis) as factors, referring to the procedures of a previous study [51], after confirming normality using the Shapiro–Wilk tests. Using three-way ANOVAs, the Mauchly’s sphericity tests were performed for effects with ≥  2 degrees of freedom with significance. If significant, the correction tests with the Greenhouse–Geisser adjustment coefficient for degrees of freedom were performed. Next, the simple interaction test was conducted to analyze the second-order interactions that are significant. If the simple interaction showed significance, the simple–simple main effect test was then performed. If the simple–simple main effect of BS was significant, the corresponding *t*-test was performed using multiple comparisons with the Benjamini-Hochberg method. Statistical analyses were performed in R (version 4.1.1). The validity of the sample size was assessed by post hoc power analysis using G * Power 3.1, and the statistical power (1 - β) was calculated. Effect sizes were reported as partial eta squared (η_p_^2^) for ANOVA results and Cohen’s d for post hoc pairwise comparisons following significant interactions in the three-way ANOVA. A p-value of <  0.05 was considered statistically significant.

## Results

### Characteristics of the participants

The participants’ mean ( ± standard deviation [SD]) value for age, height, and weight were 26 ( ± 3) years, 160.3 ( ± 6.9) cm, and 52.5 ( ± 6.8 kg), respectively. The mean ±  2 SD of height and weight for Japanese aged 20–29 years was 164.5 ±  12.2 cm and 59.8 ±  21.4 kg, respectively, calculated from data (https://www.nibiohn.go.jp/eiken/kenkounippon21/en/eiyouchousa/keinen_henka_shintai.html) reported by the National Institute of Health and Nutrition, a research institute in Japan. Therefore, the participants in this study were of average build for the Japanese population. Further, none of the participants had a history of central nervous system or orthopedic disease.

### General parameters of gait

**Fig 3:**
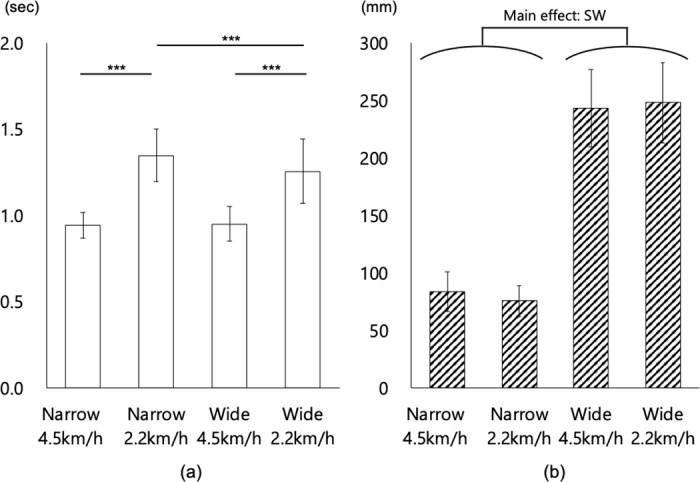
Mean data of stride time and step width under each measurement condition. (a) Stride time (b) Step width. Bars reflect mean values, and error bars represent ±  SD. *** =  p < 0.001. In the figures, results of simple main effects are shown when an interaction was observed, and a main effect is shown when no interaction was observed.

Fig 3 (and S1 Table) showed the results of the general parameters of gait. The results of the statistical analysis are presented in Table 1 and 2.

**Table 1:** Statistical results for stride time.

(a) Result of a two-way ANOVA								
Source	SS	df	MS	F-ratio	p-value	η_p_^2^	1 - β	
Factor: SW	0.03	1	0.03	9.82	0.006	0.38	0.74	**
Factor: SP	2.12	1	2.12	221.66	0.000	0.93	1	***
SW x SP	0.04	1	0.04	20.38	0.000	0.56	0.97	***
**(b) Result of the simple main effect**								
**Condition**	**SS**	**df**	**MS**	**F-ratio**	**p-value**	**η_p_^2^**	**1 - β**	
SW in Slow	0.07	1	0.07	22.70	0.000	0.59	0.95	***
SP in NB	1.38	1	1.38	143.68	0.000	0.90	1	***
SP in WB	0.79	1	0.79	82.24	0.000	0.84	1	***

SS; Sum of squares / df; degrees of freedom / MS; Mean squares / η_p_^2^; partial η^2^

SW; Step width / SP; Gait speed

NB; Narrow base / WB; Wide base / Slow; 2.2km/h

**; p<0.01/

***; p<0.001

**Table 2:** Statistical results for step width.

Source	SS	df	MS	F-ratio	p-value	η_p_^2^	1 - β	
Factor: SW	467628.84	1	467628.84	444.89	0.000	0.97	1	***
Factor: SP	33.61	1	33.61	0.08	0.776	0.01	0.06	ns
SW x SP	654.07	1	654.07	2.30	0.149	0.13	0.22	ns

SS; Sum of squares / df; degrees of freedom / MS; Mean squares / η_p_^2^; partial η^2^

SW; Step width / SP; Gait speed

ns; non-significant/

***; p<0.001

#### Stride time.

The first two-way ANOVA revealed the main effect of SW (p =  0.01) and SP (p <  0.01). That is, the participants with NB and a normal gait speed had a longer stride time than those with WB and a slow gait speed. Further, there was also an interaction between SW x SP (p <  0.01), which indicated that the simple main effect of SW was significant in participants with a slow gait speed (adjusted p <  0.01). The simple main effect of SP was significant in participants with NB (adjusted p <  0.01) and WB (adjusted p =  0.00).

#### Step width.

The first two-way ANOVA revealed the main effect of SW (p =  0.00). That is, the participants with NB had a smaller step width than those with WB.

### Parameters related to head and trunk control

#### Mean DIS.

**Fig 4:**
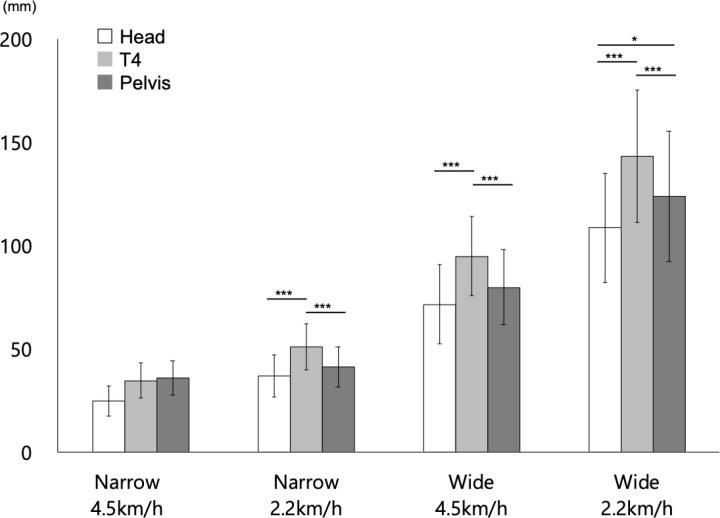
Mean data of mediolateral displacement (DIS) under each measurement condition. Bars reflect mean values, and error bars represent ±  SD. *  =  p < 0.05, and *** =  p < 0.001. In this figure, only the results of multiple comparisons between body segments within each condition are shown as asterisks. See the main text for other statistical results for main effects, simple–simple main effects, and simple main effects.

Fig 4 (and S2 Table (a)) showed the results of the general parameters of gait. The results of the statistical analysis are presented in Table 3.

**Table 3:** Statistical results for Mean DIS.

**(a) Result of a three-way ANOVA**																				
														**Mauchly’s test of sphericity**		**Greenhouse-Geisser**				
**Source**	**SS**				**df**		**MS**	**F-ratio**	**p-value**	**η**_**p**_^**2**^			**1 – β**	**W**	**p-value**	**ε**		**p-value**		
Factor: SW	224307.71				1	224307.71		188.57	0.000	0.92			1	-	-	-		-		***
Factor: SP	38014.38				1	38014.38		92.66	0.000	0.85			1	-	-	-		-		***
Factor: BS	14324.43				2	7162.22		30.21	0.000	0.65			1	0.45	0.003	0.65		0.000		***
SW x SP	13006.74				1	13006.74		46.64	0.000	0.75			1	-	-	-		-		***
SW x BS	2645.77				2	1322.89		13.67	0.000	0.46			0.99	0.34	0.000	0.60		0.000		***
SP x BS	701.49				2	350.75		14.30	0.000	0.47			0.99	0.90	0.453	-		-		***
SW x SP x BS	404.33				2	202.17		17.29	0.000	0.52			0.99	0.88	0.375	-		-		***
**(b) Result of the simple interaction effect**																				
**Source**		**SS**		**df**				**MS**			**F-ratio**				**adjusted p-value**	**η**_**p**_^**2**^				
SW x SP at Head		2631.38		1				2631.38			9.44				0.007	0.37				**
SW x SP at T4		4426.12		1				4426.12			15.87				0.002	0.50				**
SW x SP at Pelvis		6353.58		1				6353.58			22.78				0.000	0.59				***
SW x BS at Normal		1253.95		2				626.98			6.48				0.005	0.29				**
SW x BS at Slow		1796.16		2				898.08			9.28				0.001	0.37				**
SP x BS at NB		516.27		2				258.13			10.52				0.000	0.40				***
SP x BS at WB		589.56		2				294.78			12.01				0.000	0.43				***
**(c) Result of simple-simple main effects**																				
**Source**		**SS**			**df1**			**df2**				**MSE**			**F-ratio**		**adjusted** **p-value**			
SW at Normal & Head		18741.07			1			16				1189.50			15.76		0.002			**
SW at Normal & T4		30718.23			1			16				1189.50			25.82		0.000			***
SW at Normal & Pelvis		16437.88			1			16				1189.50			13.82		0.003			**
BS at NB & Normal		1281.83			2			32				237.07			2.70		0.094			#
BS at WB & Normal		4663.21			2			32				237.07			9.84		0.000			***
SW at Slow & Head		43866.32			1			16				1189.5			36.88		0.000			***
SW at Slow & T4		72550.77			1			16				1189.5			60.99		0.000			***
SW at Slow & Pelvis		58050.30			1			16				1189.5			48.80		0.000			***
BS at NB & Slow		1797.42			2			32				237.07			3.79		0.041			*
BS at WB & Slow		10333.57			2			32				237.07			21.79		0.000			***
SP at NB & Head		1247.63			1			16				410.28			3.04		0.107			ns
SP at NB & T4		2288.67			1			16				410.28			5.58		0.041			*
SP at NB & Pelvis		254.45			1			16				410.28			0.62		0.443			ns
SP at WB & Head		11635.23			1			16				410.28			28.36		0.000			***
SP at WB & T4		20143.09			1			16				410.28			49.10		0.000			***
SP at WB & Pelvis		16557.89			1			16				410.28			40.36		0.000			***
**(d) Result of multiple comparisons**																				
			**Head**												**T4**					
			**p-value**		**Cohen’s d**			**1 - β**							**p-value**		**Cohen’s d**		**1 - β**	
T4 at NB & Normal			0.000		-1.71			0.99				***			-		-		-	-
Pelvis at NB & Normal			0.000		-1.10			0.99				***			0.525		-0.16		0.10	ns
T4 at WB & Normal			0.000		-1.74			1				***			-		-		-	-
Pelvis at WB & Normal			0.146		-0.37			0.30				ns			0.000		1.23		0.98	***
T4 at NB & Slow			0.000		-1.94			0.99				***			-		-		-	-
Pelvis at NB & Slow			0.150		-0.37			0.29				ns			0.000		1.07		0.99	***
T4 at WB & Slow			0.000		-2.89			1				***			-		-		-	-
Pelvis at WB & Slow			0.012		-0.69			0.76				*			0.000		1.20		0.99	***

SS; Sum of squares / df; degrees of freedom / MSE; Mean squares error / η_p_^2^; partial η^2^

SW; Step width / SP; Gait speed / BS; Body segments

NB; Narrow base / WB; Wide base / Normal; 4.5km/h / Slow; 2.2km/h

ns; non-significant/ #; p<0.10/

*; p<0.05/

**; p<0.01/

***; p<0.001

The first three-way ANOVA indicated the main effects of SW (p <  0.01), SP (p <  0.01), and BS (p <  0.01). That is, the participants with NB and a normal gait speed had a lower DIS than those with WB and a slow gait speed.

The first-order interactions between SW x SP (p <  0.01), SW x BS (p <  0.01), and SP x BS (p <  0.01), and the second-order interactions among SW x SP x BS (p <  0.01) were significant. Hence, the second-order interactions were analyzed. Results showed that SW x SP at the head (adjusted p =  0.01), SW x SP at the T4 level (adjusted p <  0.01), SW x SP at the pelvis (adjusted p <  0.01), SW x BS under normal gait speed (adjusted p =  0.01), SW x BS under slow gait speed (adjusted p <  0.01), SP x BS under NB (adjusted p <  0.01), and SP x BS under WB (adjusted p <  0.01) were significant.

Further, based on the simple–simple main effect tests, the simple–simple main effect of SW in the head (normal: adjusted p <  0.01/slow: adjusted p <  0.01), T4 level (normal: adjusted p <  0.01/slow: adjusted p <  0.01), and pelvis (normal: adjusted p <  0.01/slow: adjusted p <  0.00) was significant in the participants with normal and slow gait speeds. Moreover, it was smaller in the participants with NB than in those with WB for all conditions and body segments. The simple main effect of SP was significant only in the T4 level (adjusted p =  0.04) in the participants with NB. Further, it was smaller in the participants with a normal gait speed than in those with a slow gait speed. Meanwhile, it was significant in the head (adjusted p <  0.01), T4 level (adjusted p <  0.01), and pelvis (adjusted p <  0.01) in the participants with WB. Moreover, it was smaller in the participants with a normal gait speed than in those with a slow gait speed for all body segments.

The simple–simple main effect of BS was significant only under slow gait speed conditions (adjusted p =  0.04) in the participants with NB. Further, it was significant under normal gait speed conditions (adjusted p <  0.01) and slow gait speed conditions (adjusted p <  0.01) in the participants with WB. Multiple comparisons with the corresponding *t*-tes*t*s revealed that the DIS of the T4 level was significantly larger than that of the head (adjusted p <  0.01) and pelvis (adjusted p <  0.01) in the participants with NB and a slow gait speed. The DIS of the T4 level was significantly larger than that of the head (adjusted p <  0.01) and pelvis (adjusted p <  0.01) in the participants with WB and a normal gait speed. Further, in the participants with WB and a slow gait speed, the DIS of the T4 level was significantly larger than that of the head (adjusted p <  0.01) and pelvis (adjusted p <  0.01). Further, the DIS of the pelvis was significantly larger than that of the head (adjusted p =  0.01).

#### Mean %DIS.

**Fig 5:**
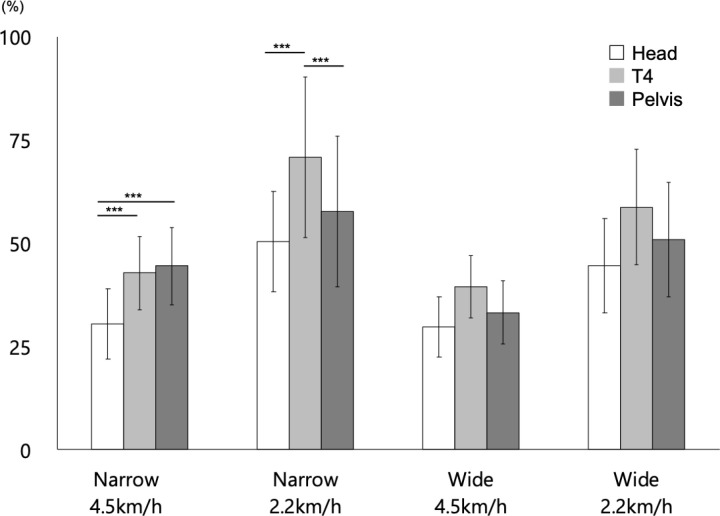
Mean data of relative magnitude of DIS to step width (%DIS) under each measurement condition. Bars reflect mean values, and error bars represent ±  SD. *** =  p < 0.001. In this figure, only the results of multiple comparisons between body segments within each condition are shown as asterisks. See the main text for other statistical results for main effects, simple–simple main effects, and simple main effects.

Fig 5 (and S2 Table (b)) showed the results of the general parameters of gait. The results of the statistical analysis are presented in Table 4.

**Table 4:** Statistical results for Mean %DIS.

**(a) Result of a three-way ANOVA**																								
																	**Mauchly’s test of sphericity**		**Greenhouse-Geisser**					
**Source**	**SS**			**df**			**MS**	**F-ratio**			**p-value**	**η**_**p**_^**2**^			**1 - β**		**W**	**p-value**	**ε**		**p-value**			
Factor: SW	2282.98			1			2282.98	15.04		0.001		0.48			1		-	-	-		-			**
Factor: SP	18071.67			1			18071.67	82.40		0.000		0.84			1		-	-	-		-			***
Factor: BS	6861.03			2			3430.52	29.27		0.000		0.65			1		0.58	0.018	0.71		0.000			***
SW x SP	124.99			1			124.99	2.03		0.173		0.11			0.86		-	-	-		-			ns
SW x BS	301.79			2			150.90	3.88		0.031		0.20			0.99		0.40	0.001	0.62		0.055			#
SP x BS	626.39			2			313.20	15.13		0.000		0.49			1		0.88	0.368	-		-			***
SW x SP x BS	385.44			2			192.72	16.57		0.000		0.51			1		0.84	0.259	-		-			***
**(b) Result of the simple interaction effect**																								
**Source**		**SS**				**df**		**MS**				**F-ratio**					**adjusted p-value**		**η**_**p**_^**2**^					
SW x SP at Head		117.24		1				117.24				1.90					0.218		0.11			ns		
SW x SP at T4		310.61		1				310.61				5.05					0.091		0.24			ns		
SW x SP at Pelvis		82.58		1				82.58				1.34					0.264		0.08			ns		
SW x BS at Normal		512.50		2				256.25				6.59					0.014		0.29			*		
SW x BS at Slow		174.73		2				87.36				2.25					0.181		0.12			ns		
SP x BS at NB		921.38		2				460.69				22.25					0.000		0.58			***		
SP x BS at WB		90.45		2				45.23				2.18					0.181		0.12			ns		
**(c) Result of simple-simple main effects**																								
**Source**		**SS**		**df1**				**df2**				**MSE**					**F-ratio**		**adjusted** **p-value**					
SW at Normal & Head		4.46		1				16				151.80					0.03		0.866			ns		
SW at Normal & T4		96.71		1				16				151.80					0.64		0.466			ns		
SW at Normal & Pelvis		1081.14		1				16				151.80					7.12		0.027			*		
BS at NB & Normal		2021.07		2				32				117.20					8.62		0.005			**		
BS at WB & Normal		825.44		2				32				117.20					3.52		0.055			#		
SP at NB & Head		3416.17		1				16				219.31					15.58		0.005			**		
SP at NB & T4		6624.39		1				16				219.31					30.21		0.000			***		
SP at NB & Pelvis		1482.08		1				16				219.31					6.76		0.028			*		
BS at NB & Slow		3579.91		2				32				117.20					15.27		0.000			***		
**(d) Result of multiple comparisons**																								
			**Head**											**T4**										
			**p-value**		**Cohen’s d**				**1 - β**					**p-value**		**Cohen’s d**				**1 - β**				
T4 at NB & Normal			0.000		-1.80				0.99				***	-		-				-			-	
Pelvis at NB & Normal			0.000		-1.11				0.99				***	0.481		-0.17				0.10			ns	
T4 at NB & Slow			0.000		-1.52				0.99				***	-		-				-			-	
Pelvis at NB & Slow			0.077		-0.46				0.43				#	0.000		1.47				0.99			***	
T4 at WB & Normal			0.000		-1.95				1				***	-		-				-			-	
Pelvis at WB & Normal			0.151		-0.37				0.28				ns	0.001		0.99				0.97			**	

SS; Sum of squares / df; degrees of freedom / MSE; Mean squares error / η_p_^2^; partial η^2^

SW; Step width / SP; Gait speed / BS; Body segments

NB; Narrow base / WB; Wide base / Normal; 4.5km/h / Slow; 2.2km/h

ns; non-significant/ #; p<0.10/

*; p<0.05/

**; p<0.01/

***; p<0.001

The first three-way ANOVA revealed the main effects of SW (p <  0.01) and SP (p <  0.01). The %DIS of the participants with NB was larger than that of the participants with WB. Moreover, the %DIS of the participants with a normal gait speed was smaller than that of the participants with a slow gait speed.

The main effect of BS (p <  0.01), the first-order interaction between SP x BS (p <  0.01), and the second-order interaction between SW x SP x BS (p <  0.01) were significant. Hence, the second-order interactions were analyzed. Results showed that the simple interactions between SW x BS (adjusted p =  0.01) in the participants with a normal gait speed and SP x BS (adjusted p <  0.01) in the participants with NB were significant.

Further, based on the simple–simple main effect tests, the simple–simple main effect of SW in the pelvis (adjusted p =  0.03) in the participants with a normal gait speed was significant. The %DIS of the participants with NB was greater than that of the participants with WB. The simple–simple main effect of SP for the head (adjusted p <  0.00), T4 level (adjusted p <  0.01), and pelvis (adjusted p =  0.03) in the participants with NB was significant. Thus, the %DIS for all body segments in the participants with the normal gait speed was greater than that in the participants with a slow gait speed.

The simple–simple main effect of BS was significant under normal gait speed conditions (adjusted p <  0.01) and slow gait speed conditions (adjusted p <  0.01) in the participants with NB. Further, according to multiple comparisons with the corresponding *t*-tes*t*s, the %DIS for the head was significantly smaller than that for the T4 level (adjusted p <  0.01) and pelvis in the participants with NB and a normal gait speed. In addition, the %DIS for the T4 level was significantly larger than that for the head (adjusted p <  0.01) and pelvis (adjusted p <  0.01) in the participants with NB and a slow gait speed.

#### CV-%DIS.

**Fig 6:**
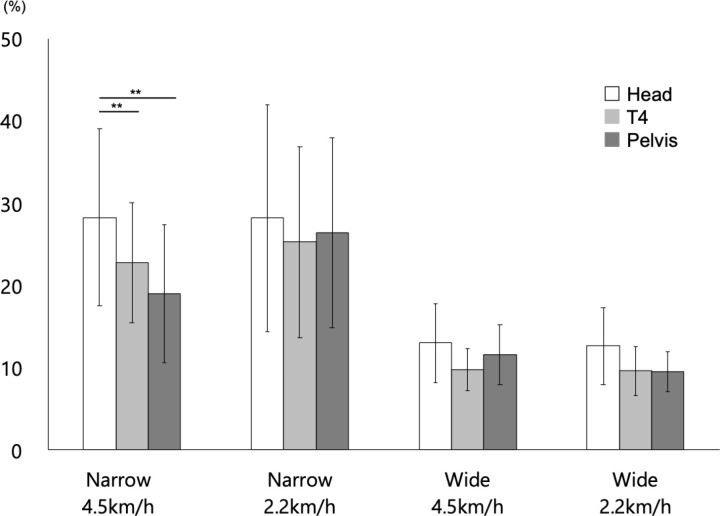
Variability of %DIS (CV-%DIS) under each measurement condition. Bars reflect mean values, and error bars represent ±  SD. *  =  p < 0.05, ** =  p < 0.01, and *** =  p < 0.001. In this figure, only the results of multiple comparisons between body segments within each condition are shown as asterisks. See the main text for other statistical results for the main effects, simple–simple main effects, and simple main effects.

Fig 6 (and S2 Table (c)) showed the results of the general parameters of gait. The results of the statistical analysis are presented in Table 5.

**Table 5:** Statistical results for CV-%DIS.

**(a) Result of a three-way ANOVA**																																		
																						**Mauchly’s test of sphericity**					**Greenhouse-Geisser**							
**Source**		**SS**	**df**		**MS**				**F-ratio**			**p-value**				**η**_**p**_^**2**^			**1 - β**			**W**		**p-value**			**ε**		**p-value**					
Factor: SW		9941.34	1		9941.34				96.36			0.000				0.86			1			-		-			-		-				***	
Factor: SP		75.56	1		75.56				0.43			0.523				0.03			0.29			-		-			-		-				ns	
Factor: BS		650.55	2		325.27				15.26			0.000				0.49			1			0.77		0.144			0.81		0.000				***	
SW x SP		216.84	1		216.84				1.32			0.268				0.08			0.71			-		-			-		-				ns	
SW x BS		90.98	2		45.49				5.91			0.007				0.27			0.99			0.99		0.934			0.99		0.007				**	
SP x BS		72.87	2		36.43				1.44			0.251				0.08			0.71			0.55		0.012			0.69		0.252				ns	
SW x SP x BS		192.90	2		96.45				6.87			0.003				0.30			0.99			0.77		0.140			0.81		0.006				**	
**(b) Result of the simple interaction effect**																																		
**Source**	**SS**						**df**				**MS**						**F-ratio**						**adjusted p-value**					**η**_**p**_^**2**^						
SW x SP at Head	0.28						1				0.28						0.00						0.967					0.00				ns		
SW x SP at T4	30.23						1				30.23						0.18						0.832					0.01				ns		
SW x SP at Pelvis	379.22						1				379.22						2.31						0.346					0.13				ns		
SW x BS at Normal	274.07						2				137.04						17.81						0.000					0.53				***		
SW x BS at Slow	9.80						2				4.90						0.64						0.832					0.04				ns		
SP x BS at NB	248.50						2				124.25						4.92						0.048					0.24				*		
SP x BS at WB	17.26						2				8.63						0.34						0.832					0.02				ns		
**(c) Result of simple-simple main effects**																																		
**Source**						**SS**				**df1**			**df2**		**MSE**						**F-ratio**					**adjusted** **p-value**								
SW at Normal & Head						1977.4439				1			16		103.164						19.1679					0.0015					**			
SW at Normal & T4						1435.7263				1			16		103.164						13.9169					0.0049					**			
SW at Normal & Pelvis						471.7776				1			16		103.164						4.5731					0.1103					ns			
BS at NB & Normal						737.2601				2			32		21.314						17.2956					0.0001					***			
BS at WB & Normal						87.9784				2			32		21.314						2.0639					0.2296					ns			
SP at NB & Head						0.0519				1			16		177.02						0.0003					0.9866					ns			
SP at NB & T4						52.0632				1			16		177.02						0.2941					0.7934					ns			
SP at NB & Pelvis						470.5825				1			16		177.02						2.6584					0.2178					ns			
BS at NB & Slow						73.1963				2			32		21.314						1.7171					0.2847					ns			
**(d) Result of multiple comparisons**																																		
				**Head**																**T4**														
				**p-value**				**Cohen’s d**						**1 - β**						**p-value**					**Cohen’s d**					**1 - β**				
T4 at NB & Normal				0.009				0.77						0.85				**		-					-					-				-
Pelvis at NB & Normal				0.002				1.00						0.97				**		0.061					0.49					0.48				#

SS; Sum of squares / df; degrees of freedom / MSE; Mean squares error / η_p_^2^; partial η^2^

SW; Step width / SP; Gait speed / BS; Body segments

NB; Narrow base / WB; Wide base / Normal; 4.5km/h / Slow; 2.2km/h

ns; non-significant/ #; p<0.10/

*; p<0.05/

**; p<0.01/

***; p<0.001

The first three-way ANOVA revealed the main effect of SW (p <  0.01). Hence, the CV-%DIS in the participants with NB was larger than that in the participants with WB.

The main effect of BS (p <  0.01), the first-order interaction between SW x BS (p =  0.01), and the second-order interaction between SW x SP x BS (p <  0.01) were significant. Therefore, the second-order interactions were analyzed. Results showed that the simple interactions between SW x BS in the participants with a normal gait speed (adjusted p <  0.01) and SP x BS in those with NB (adjusted p =  0.04) were significant.

Further, the simple–simple main effect of SW for the head (adjusted p <  0.01) and T4 level (adjusted p <  0.01) was significant in the participants with a normal gait speed. Moreover, the CV-%DIS of the participants with NB was greater than that of the participants with WB.

The simple–simple main effect of SP was not significant in either case. The simple–simple main effect of BS was significant only in the participants with NB and a normal gait speed (adjusted p <  0.01). Hence, multiple comparisons with the corresponding *t*-*t*ests showed that the CV-%DIS for the head was significantly larger than that for the T4 level (adjusted p =  0.01) and pelvis (adjusted p <  0.01) in the participants with NB and a normal gait speed.

## Discussion

This study established four conditions combining the step width and gait speed to examine how the mediolateral behaviors of the head and trunk (thoracic and pelvic regions) are controlled during gait. Some indices including stride time, step width, DIS (the mediolateral displacement) of the head and trunk (the thoracic and pelvic regions), %DIS (the ratio of the displacement of each segment relative to the width of the BOS), and CV-%DIS (the variability of that ratio) were used. For indices related to segments with high mass ratios, we assessed the overall trends of segments with high mass ratios based on the main effects of step width and gait speed. Further, the associations between segments with high mass ratios were examined based on the simple–simple main effects and multiple comparison results.

In the gait conditions of this study, the step width and the gait speed affected the length of stride time, with the influence of step width occurring only under slow gait speed conditions. However, the influence of gait speed occurred regardless of the step width, thereby indicating the stronger effect of gait speed. In particular, longer stride times were observed under slower gait speed conditions. Meanwhile, shorter stride times were observed under normal gait speed conditions. This finding is consistent with that of previous studies [52,53,54]. Moreover, it is attributed to the faster speeds maintaining the consistency of the gait cycle and eliciting an automated, regular rhythm of the gait [55]. The size of the step width was influenced by the step width, but not by gait speed, thereby supporting previous findings [28,54]. In addition, this result confirmed that the conditions set for the step width in this study were adequately achieved.

First, the overall tendency of high mass ratio segments was evaluated based on the main effects of step width and gait speed.

The main effect of SW showed that step width influenced DIS, %DIS, and CV-%DIS. In particular, if the step width was narrow, the body segments with high mass ratios had a shorter DIS (Fig 4), larger %DIS (Fig 5), and higher CV-%DIS (Fig 6). Therefore, the whole body segment with a high contribution to COM under narrow BOS conditions is more likely to have a high variability. However, the mediolateral direction behavior was small. Thus, the body segments with higher mass ratios were more likely to be finely and coordinately controlled if the step width is narrow. Hence, narrowing the step width, or BOS, may have induced a more advanced control strategy in body segments with high mass ratios. Conversely, if the step width was wide, the body segments with high mass ratios had a longer DIS (Fig 4), smaller %DIS (Fig 5), and lower CV-%DIS (Fig 6). Therefore, the whole body segment with a high contribution to COM under wide BOS conditions was more likely to have a low variability. Nevertheless, the mediolateral direction behavior was large. Thus, unlike if the step width was narrow, the body segments with high mass ratios were uniformly controlled without fine control if the step width was wide. Therefore, the body segments with high mass ratios could control balance in a regular and simple manner during gait. These results can be one of the reasons why in actual rehabilitation clinical situations, a wider step width can be achieved by compensating for the impaired coordination and instability of the trunk [56], particularly in individuals with ataxia [57], those who present with postural control disorders and trunk dysfunction caused by nervous system conditions such as stroke [58] and cerebral palsy [59]. Hence, widening the step width, or BOS, may have induced a simpler control strategy in the body segments with high mass ratios during gait. Therefore, a narrow step width induces atypical and advanced control of the body segments with high mass ratios. In contrast, a wide step width induces a typical and simpler control. Consequently, adjusting the step width indirectly adjusts the difficulty of controlling the body segments with high mass ratios and the load on the control system of the gait.

Next, based on the main effect of SP, gait speed influences DIS and %DIS, but not CV-%DIS. Gait speed strongly influences various spatiotemporal parameters such as cadence and step length, lower limb joint movements, and vertical ground reaction forces [60]. The current study revealed that gait speed also affected the behavior of the body segments with high mass ratios. In particular, in the standard gait speed condition, the body segments with high mass ratios exhibited a shorter DIS and smaller %DIS. Results showed that the body segments with high mass ratios were controlled by a high stability under normal gait speed conditions. According to previous studies, the MOS of COM is larger [61]. Meanwhile, the mediolateral amplitudes of the head, shoulders, and pelvis are smaller under normal than under slow gait speed conditions [62]. Therefore, the stability of COM is affected by the stability of the body segments with high mass ratios. Conversely, under slow gait speed conditions, the body segments with high mass ratios exhibited a longer DIS and larger %DIS. This result can indicate that the body segments with high mass ratios are controlled by a low stability under slow gait speeds. Therefore, facilitating motor and sensory input is important in maintaining mediolateral balance and may alter the kinematic properties of the trunk [63].

In the following sections, the association between the body segments and high mass ratios was examined based on the simple–simple main effects and multiple comparisons. Based on the results of multiple comparisons, the thorax region had a significantly higher DIS (Fig 4), except for the narrow step width and fast gait speed, than the head and pelvic region. However, there was no significant difference in terms of narrow step width and fast gait speed between the head, thorax, and pelvic region. In cases of narrow step width and slow gait speed, the thorax region had a significantly higher %DIS (Fig 5) than the head and pelvic regions. Conversely, in cases of narrow step width and fast gait speed, the head had a significantly higher %DIS than the thorax and pelvic regions. Notably, there was no significant difference between the head, thorax region, and pelvic regions if the step width was wide. In cases of narrow step width and fast gait speed, the head presented with a significantly higher CV-%DIS (Fig 6) than the thorax and pelvic regions. However, in other conditions, there was no significant difference between the head, thorax, and pelvic regions in terms of CV-%DIS.

In the kinematics of the head during gait, the lateral flexion is lower in normal gait speed conditions than in slow speed gait conditions [64]. In addition, the mechanisms of involuntary stabilization and positioning of the head suppress high-frequency oscillations, controlling acceleration to a minimum, particularly in the lateral direction [59]. Therefore, the strategies for stabilizing the head can be achieved by adjusting multiple sensory inputs to the sensory receptors of the head [65], such as visual input [17]. The current study revealed that the DIS (Fig 4) of the head was spatially small regardless of step width and gait speed except in narrow normal conditions. Further, the %DIS of the head (Fig 5) was significantly small only in narrow step widths, thereby indicating that the head movements are controlled by a small range of the frontal plane. In addition, according to the significantly higher CV-%DIS in the narrow step width at a normal gait speed, the control was more fine-tuned in the position of the head relative to the feet (Fig 6). Adjustments in the association between the sensory inputs to the sensory receptors in the feet and head may have fine-tuned the relative position of the head with respect to the feet during gait. This stabilization strategy may have been implemented in conjunction with the fluctuating position of the feet with each step.

The mediolateral displacement of the thorax located at the upper part of the trunk and one of the high mass ratio segments are the most significant on the stance side [18], thereby maintaining alignment from the head to the trunk in the sagittal plane by increasing the lateral tilt angle compared with that in the pelvis [16]. This is believed to achieve head stability, with control of high-amplitude vibrations occurring in the trunk before reaching the head or neck [65]. In maintaining head stability, the trunk, particularly the thorax, plays an important role in providing stable support for higher segments [65,66]. In this study, the thoracic regions had a significantly larger DIS than other segments except for the narrow normal condition and a significantly larger %DIS, particularly at slow speed and narrower step widths (Figs 4, 5). These results indicated that the thoracic region, as the high mass ratios segment, was controlled within a larger range than the other segments in the frontal plane in all conditions except for narrow step width and normal speed gait, which is considered a more challenging control. Moreover, the thoracic region may be significantly important in maintaining trunk control for balance on the frontal plane during gait [60]. The kinematics of the thoracic region in this study support a previous study, and results showed that the trunk movement pattern in the frontal plane must be remarkable [17]. In addition, the thoracic region, which has a high mass ratio, was significantly larger in the mediolateral direction under the wide step width conditions. Meanwhile, there was no significant difference in the variability of segmental movement compared to other segments. Therefore, control in the thoracic region may play an important role in damping and stabilizing head oscillations by affecting the maintenance of trunk balance in the frontal plane. Furthermore, the minimum walking speed for the elderly has been reported to be 2.2-2.5 km/h [42], and a study of treadmill walking for stroke patients set the walking speed at 2.3 km/h [67], which is the low speed set in this study. Therefore, it is believed that by understanding the state of coordinated segmental control of the head to the trunk at 2.2 km/h in healthy young adults, suggestions for subjects with reduced segmental control capabilities can be provided. The study results showed that at slow speeds with a narrow base, the distance moved by the thorax segment standardized by step width was significantly greater than that of the head and pelvis. The walking speed decreases and the step width widens in individuals with ataxic gait disorders, elderly, and patients with spastic cerebral stroke [68,69]. The study findings emphasize the need for segmental control of the thoracic region relative to the head and pelvis to coordinate segmental control in the range of the narrow base, which is composed of the foot. In contrast, the limited range of motion and reduced ability to coordinate control from the head to the trunk may be related to walking difficulty on a narrow base and the reduced walking speed. A walking speed of 2.2 km/h is also considered to be a speed that increases the risk of falls in the elderly; therefore, improving walking stability at low speeds is important [44]. To improve walking stability at low speeds, training programs that improve the coordination from the head to the trunk with a particular focus on thoracic segmentation may be effective.

The findings of this study revealed the potential to estimate the load for control systems and gait strategies based on the mediolateral range of displacement and variability in the segmented control of the head and thoracic and pelvic regions. Consequently, when evaluating gait in MOS with impaired head, thorax, and pelvic control, it may be effective to consider the influence of BOS size and gait speed on at least each of the three segments. Moreover, these findings are likely to contribute to a greater understanding of the fundamental issues of balance control in walking and may assist in developing effective clinical treatment programs. For example, effective clinical treatment programs for individuals with conditions such as ataxia [57], stroke [58], and cerebral palsy [59], who have wide step widths, may need to consider the effect of difficulties in segmental control from the head to the trunk.

This study has several limitations. First, the sample size was smaller than that of other studies [26], and the fact that all participants were healthy young adults may limit the generalizability of the results. Therefore, to apply the results clinically, additional verification in older adults and patients with stroke, who have been reported to have reduced trunk range of motion during walking, is essential. Second, due to the limitations of the measurement devices and settings, the number of markers was limited, and the study could not analyze the sagittal and horizontal planes or to consider the relationship with joint motions in the lower extremities. Furthermore, this study only used the results of the kinematic aspect of the motion analysis and was unable to verify the detailed mechanisms, such as through kinetic analysis, including ground reaction forces and electromyography, or neurophysiological analysis. Moreover, while treadmill walking, the measurement condition used in this study has the great advantage of being able to measure repeated and continuous walking; therefore, further verification is necessary regarding its equivalence to the results of walking normally. Finally, in this study, we analyzed coordinated segmental control based on movement variability occurring in each segment from the head to the trunk; however, we were unable to provide specific criteria for determining whether the CV-%DIS value, which is the index used in this study, was “high” or “low.” To verify the changes in the patterns and strategies of coordinated control of the segmentation of the head to trunk in walking, analyzing the data in more detail is necessary, including nonlinear analysis, under various measurement conditions and for different subject types.

## Conclusion

This study assessed the mediolateral behaviors of the head, thorax, and pelvis during gait under various conditions of step width and gait speed. The following results were obtained:

Step width influence: A narrow step width prompted more coordinated and fine-tuned control in body segments with high mass ratios, resulting in a smaller displacement (DIS), larger relative displacement (%DIS), and higher variability (CV-%DIS). Conversely, a wide step width led to simpler, more uniform control strategies, with a longer DIS, smaller %DIS, and lower CV-%DIS, indicating a less challenging control of balance.

Gait speed influence: The standard gait speed had a greater stability with a shorter DIS and a smaller %DIS in high mass body segments, thereby indicating efficient control mechanisms. A slower speed resulted in a greater DIS and larger %DIS, thereby highlighting a decreased stability and altered control dynamics to maintain balance.

Intersegment relationships: The head had highly controlled movements with minimal displacement and increased fine-tuning, particularly at narrow step widths and standard speeds. The thorax was significantly important in maintaining trunk stability, operating within a larger range of mediolateral displacement compared with the head and pelvis, particularly under challenging gait conditions.

The current study underscores the significant impact of both step width and gait speed on the control and stability of high mass body segments during gait. Thus, narrow step widths require advanced control strategies. Meanwhile, wide step widths can promote simpler, compensatory mechanisms, particularly relevant in clinical contexts.

These insights have vital implications for clinical treatments that aim to improve balance and gait stability in populations with coordination and stability impairments, such as those with ataxia, stroke, and cerebral palsy. The indices used in this study are calculated from the distance moved by each segment during treadmill walking. They do not require complicated analysis. Therefore, they are easy to apply it to the clinical evaluation of coordinated control of the head-to-trunk segment during walking. Furthermore, we believe that the study results on healthy young adults will be helpful as basic data for the clinical evaluation of coordinated control of segmental movement from the head to the trunk during walking. Therefore, we conclude that the study results help analyze the factors that cause reduced stability of walking, such as the effects of aging, spinal lesions, and stroke. Future research should expand on these findings by including diverse populations, thereby enhancing measurement techniques, and exploring specific control patterns and balance strategies.

## Supporting information

S1 File Dataset of all parameters. (XLSX)

S1 Table Mean data of stride time and step width in each condition. (DOCX)

S2 Table Mean data of parameters related to head and trunk control in each condition. (DOCX)
