# Supplementary material for: Effects of step width and gait speed on the variability of mediolateral control in the head and trunk during gait
Source: PLoS One. 2025 Apr 21;20(4):e0320652. doi: 10.1371/journal.pone.0320652 (PMC12011241; doi:10.1371/journal.pone.0320652)
Supplement: S2 Table — (DOCX) [file pone.0320652.s003.docx]

**S2 Table. Mean data of parameters related to head and trunk control in each condition.**

| (a) Mean DIS  Mean (SD) (mm) | | | | |
| --- | --- | --- | --- | --- |
|  | NB, Normal | NB, Slow | WB, Normal | WB, Slow |
| Head | 24.6 (7.4) | 36.7 (10.2) | 71.6 (19.0) | 108.5 (26.6) |
| T4 | 34.5 (8.4) | 50.9 (11.1) | 94.7 (19.2) | 143.3 (32.0) |
| Pelvic | 35.8 (8.4) | 41.3 (9.6) | 79.8 (18.3) | 123.9 (31.6) |
| (b) Mean %DIS  Mean (SD) (%) | | | | |
|  | NB, Normal | NB, Slow | WB, Normal | WB, Slow |
| Head | 30.4 (8.5) | 50.4 (12.1) | 29.7 (7.3) | 44.5 (11.4) |
| T4 | 42.8 (8.9) | 70.7 (19.4) | 39.4 (7.5) | 58.8 (14.1) |
| Pelvic | 44.5 (9.4) | 57.7 (18.2) | 33.2 (7.7) | 50.9 (13.8) |
| (c) CV-%DIS  Mean (SD) (%) | | | | |
|  | NB, Normal | NB, Slow | WB, Normal | WB, Slow |
| Head | 28.3 (10.7) | 28.2 (13.8) | 13.0 (4.8) | 12.7 (4.7) |
| T4 | 22.8 (7.3) | 25.3 (11.6) | 9.8 (2.6) | 9.6 (3.0) |
| Pelvic | 19.0 (8.4) | 26.4 (11.5) | 11.6 (3.6) | 9.6 (2.5) |

SD; Standard Deviation

NB; Narrow base / WB; Wide base / Normal; 4.5km/h / Slow; 2.2km/h
